# *KLF5* promotes the ossification process of ligamentum flavum by transcriptionally activating *CX43*

**DOI:** 10.1186/s13018-024-04702-2

**Published:** 2024-04-16

**Authors:** Hubing Guo, Lingxia Yang, Jin Liu, Liqi Chen, Yufeng Huang, Jinsong Li

**Affiliations:** 1 The First Department of Orthopaedic Surgery, The First Hospital of Tianshui, Tianshui, Gansu, 741000 China; 2 Department of Odermatology, The First Hospital of Tianshui, Tianshui, Gansu, 741000 China; 3 Department of Spine Surgery, The Third Xiangya Hospital, Central South University, No.138 Tongzipo Road, Changsha, 410013 Hunan China

**Keywords:** KLF5, Ligamentum flavum, Ossification, CX43

## Abstract

**Background:**

Ossification of ligamentum flavum (OLF) is a prevalent degenerative spinal disease, typically causing severe neurological dysfunction. Kruppel-like factor 5 (KLF5) plays an essential role in the regulation of skeletal development. However, the mechanism KLF5 plays in OLF remains unclear, necessitating further investigative studies.

**Methods:**

qRT-PCR, immunofluorescent staining and western blot were used to measure the expression of KLF5. Alkaline Phosphatase (ALP) staining, Alizarin red staining (ARS), and the expression of Runt-related transcription factor 2 (RUNX2), osteopontin (OPN), and osteocalcin (OCN) were used to evaluate the osteogenic differentiation. Luciferase activity assay and ChIP-PCR were performed to investigate the molecular mechanisms.

**Results:**

KLF5 was significantly upregulated in OLF fibroblasts in contrast to normal ligamentum flavum (LF) fibroblasts. Silencing KLF5 diminished osteogenic markers and mineralized nodules, while its overexpression had the opposite effect, confirming KLF5’s role in promoting ossification. Moreover, KLF5 promotes the ossification of LF by activating the transcription of Connexin 43 (CX43), and overexpressing CX43 could reverse the suppressive impact of KLF5 knockdown on OLF fibroblasts’ osteogenesis.

**Conclusion:**

KLF5 promotes the OLF by transcriptionally activating CX43. This finding contributes significantly to our understanding of OLF and may provide new therapeutic targets.

## Introduction

Ossification of ligamentum flavum (OLF), one of the degenerative conditions of the spine, is a widely recognized health issue [1]. It manifests predominately through the calcification of paravertebral cartilage and fibrous tissue, thereby inducing a pathological transformation process that involves various biological constituents [2, 3]. Several factors, including age, gender, ethnicity, and genetics, play significant roles in determining the incidence and severity of OLF [4, 5]. Ligamentum flavum (LF) is an integral part of the spine; hence, any abnormality in its structure poses risks to other components of the spinal cord, nerve roots, and vertebral arteries [6]. The ensuing compression often triggers a variety of debilitating symptoms, such as pain, burning sensations, and limb weakness, which significantly impede the affected individuals’ quality of life [7]. In recent times, the exploration of OLF’s molecular pathology has considerably progressed, unveiling the noteworthy role of its ligament fibroblasts [8]. These fibroblasts display an enhanced capacity for osteogenic differentiation, a key contributor to the progression of ectopic ossification [9]. However, the specificity of pathogenesis, in terms of precise molecular mechanisms, still needs to be fully comprehended.

Kruppel-like factor 5 (KLF5), encoded by the KLF5 gene, a member of the Sp1/KLF transcription factor family [10], works as a versatile transcription factor that has a robust influence on a wide array of cellular processes, including cell proliferation, differentiation, migration, and apoptosis [11, 12]. Recent research has highlighted KLF5’s significant contribution to the regulation of skeletal development by acting as a positive facilitator of osteogenesis [13, 14]. Despite these advancements, the explicit function of KLF5 in the context of OLF has not been fully elucidated, pointing to a gap in knowledge that current and future studies aim to fill.

Connexins (Cxs), known as gap junction (GJ) transmembrane proteins, play a vital role in intercellular communication. Connexin 43 (CX43), also known as GJA1, is the most extensively examined variant in the connexin protein family, predominantly due to its significant presence in cardiac, mammary, cerebral, and colorectal tissues [15, 16]. This prominence is mirrored in bone, where CX43 is the chief connexin protein, with gap junctions rich in CX43 monomers facilitating extensive intercellular connectivity among osteoblasts and osteocytes [17]. Several studies have found that CX43 affects osteoblastic differentiation [18–20]. Moreover, Chen et al. indicated that CX43 supports the osteogenic differentiation of LF fibroblasts associated with ossification [21]. Interestingly, the JASPAR database predicts a potentially instrumental role of KLF5 in serving as a transcription factor for CX43. However, the purported regulatory relationship between the two needs more empirical substantiation in the previous research.

With this background, our study aimed to unravel the potential mechanism of the KLF5-CX43 axis worked in LF ossification, which is expected to provide a theoretical groundwork conducive to improved and targeted therapeutic interventions for OLF.

## Methods

### Ligament samples collection

The LF tissue of OLF samples was collected from ten patients (aged 58–82) diagnosed with OLF in The First Hospital of Tianshui and The Third Xiangya Hospital of Central South University; four females and six males were involved in this study, while normal LF samples were obtained from seven volunteers (aged 42–68) diagnosed with spinal trauma or disc herniation, including three females and four males. Prior to participation, informed consent was acquired from all patients. The Ethics Committee of Third Xiangya Hospital, Central South University, approved this research protocol. After the surgical excursions, we immediately preserved the collected ligament tissues in liquid nitrogen, typically within a thirty-minute post-surgical window, to maintain their integrity.

### Primary LF fibroblasts culture and osteogenic differentiation

Primary LF fibroblasts were isolated via the tissue explant technique following a protocol outlined in previous literature [2]. Initially, ligament samples were cleared of non-ligamentous tissue under a dissecting microscope and then subjected to a PBS rinse. The cleaned ligament tissues were then finely diced into 0.5 mm^3^ pieces and underwent a dual PBS wash to remove residual debris. These prepared tissues were enzymatically dissociated first with 0.25% trypsin (Gibco, USA) for one hour at 37℃ and then with 200 U/mL of type I collagenase (Sigma-Aldrich, USA) for an additional four hours at the same temperature. Following digestion, the tissue fragments were cultured in DMEM (Hyclone, USA) that was fortified with 10% FBS, 100 U/mL penicillin, and 100 µg/mL streptomycin (Gibco, USA), maintained at 37 °C in a 5% CO2 humidified environment.

To induce osteogenic differentiation, we cultured fibroblasts in an osteogenic medium formulated with DMEM enriched with 10% FBS, 0.1 mM dexamethasone, 0.2 mM ascorbic acid, and 10 mM β-glycerophosphate (Sigma-Aldrich). This specialized medium supports the cells’ progression toward an osteogenic phenotype.

### RNA interference

KLF5 siRNA is purchased from Ribobio (Guangzhou, China). Fibroblasts were placed into 24-well plates and cultured in a complete medium for roughly 24 h until they reached around 60–70% confluence. Following this, these fibroblasts are transfected with the siRNA at the described concentrations, using lipofection with the agent Lipofectamine 3000.

### Lentiviral infection of LF fibroblasts

Lentiviral vectors for the overexpression of KLF5 and CX43 were sourced from Ribobio (China). Corresponding empty vectors served as negative controls (Lenti-NC). In preparation for infection, fibroblasts were plated at 5 × 10^5^ cells per well in 100 mm culture dishes to establish a suitable environment for viral infection. On the second day post-seeding, we added a 100 µl infection mixture containing 5 × 10^5^ PFU of the lentivirus directly to the culture medium of the fibroblasts. Post-infection, cells were maintained in an incubator at 37 °C with a humidified atmosphere of 5% CO2 for optimal growth conditions.

### Quantitative real-time PCR (qRT-PCR)

Total RNA was extracted from cultured fibroblasts utilizing TRIzol reagent (Invitrogen Life Technologies, Carlsbad, CA, USA), adhering to a standard protocol [22]. The purity and integrity of the extracted RNA were verified through Nanodrop 2000 (Thermo Scientific, USA). Subsequently, 1 µg of total RNA was reverse transcribed to cDNA using the HiScrip III 1st Strand cDNA Synthesis Kit (Vazyme, China). Primer Design and PCR Amplification Primers specific to the genes of interest, KLF5, and CX43, were formulated using Primer 3.0 software (ABI, USA) and synthesized by Tsingke Biotech (Beijing, China). The primer sequences were as follows: KLF5-F, 5′-ATGGAGAAGTATCTGACACCTCA-3′; KLF5-R, 5′-TCAGTTCTGGTGCCTCTTCATATG-3′; CX43-F, 5′-CTTCACTACTTTTAAGCAAAAGAG-3′; CX43-R, 5′-TCCCTCCAGCAGTTGAG-3′; GAPDH-F, 5′-ATGGTTTACATGTTCCAATATGA-3′; GAPDH-R, 5′-TTACTCCTTGGAGGCCATGTGG-3′. The qRT-PCR assays were carried out in triplicate, employing SYBR-Green Master mix (Vazyme, China) on a LightCycler 480 (Roche, USA). The amplification protocol was initiated with a 95 °C denaturation step for 30 s, followed by 40 cycles of 95 °C for 10 s, and a combined annealing/extension phase at 60 °C for 30 s. Data Analysis The quantification of mRNA levels was based on the 2^^-ΔΔCt^ method. Expression levels of the target genes were normalized using the endogenous control GAPDH.

### Immunofluorescent staining

Fibroblasts were fixed using 4% paraformaldehyde for 15 min at 4℃, followed by permeabilization with 0.1% Triton X-100 for 5 min at room temperature, then washed with PBS and blocked with 5% BSA (Solarbio, China) for 30 min at room temperature to prevent non-specific binding. After blocking, fibroblasts were incubated with a primary antibody targeting KLF5 (1:200 dilution, Abcam, USA) overnight at 4 ℃ and then incubated with Dylight Fluor-conjugated secondary antibodies for 1 h at 37 ℃. DAPI was applied to stain the cells for 5 min at room temperature to label the nucleus. Imaging Immunofluorescence signals were detected and imaged using a confocal microscope (Leica TCS SP8, GER).

### Western blot

Fibroblasts were lysed using pre-chilled lysis buffer at 4℃. The BCA assay kit (Abcam, USA) was employed to quantify the total protein content in each sample. Measured aliquots of 30 µg total protein per sample were subjected to electrophoresis on a 4-20% gradient SDS-PAGE gel and transferred onto PVDF membranes. Membranes were blocked using a solution of 5% skim milk in TBS-T and incubated with primary antibodies, all from Abcam, against KLF5 (1:200), Runt-related transcription factor 2 (RUNX2, 1:1000), osteocalcin (OCN, 1:1000), osteopontin (OPN, 1:1000), CX43 (1:1000), and β-actin (1:200) at 4℃ overnight. Following primary antibody incubation, membranes were treated with peroxidase-conjugated anti-rabbit IgG secondary antibody (1:5000, Millipore Corporation). Enhanced chemiluminescence reagent (Merck Millipore, Billerica, MA, USA) and a chemiluminescence imaging system (Bio-Rad Laboratories, Inc) were used for visualization. Protein bands were quantified using ImageJ software version 1.52 (National Institutes of Health, USA), with β-actin as the loading control.

### Alkaline phosphatase (ALP) activity assay

Approximately 10^5^ cells per well were plated into six-well plates to assess osteogenic differentiation. Upon reaching 65% confluence, the cells were subjected to an osteogenic induction medium to initiate differentiation. After seven days of osteogenic induction, cells were rinsed with PBS and fixed with 4% formaldehyde for 15 min. The detection of ALP activity was performed using an ALP staining kit (Stemgent 00–0055; Mito Biological technology Co,.LTD, Shanghai, China) following the manufacturer’s instructions, then washed with PBS and examined under a microscope to visualize ALP activity.

### Alizarin red staining (ARS)

Following the osteogenic induction of primary LF fibroblasts, the cells were fixed using 95% ethanol for 30 min to preserve cellular structures for staining. After fixation, the cells underwent two PBS washes to remove any traces of fixative. Staining was carried out with a 0.2% ARS solution (Biosharp, China) at 37 °C for 30 min to label calcium deposits indicative of mineralization. Post-staining, the fibroblasts were rinsed with distilled water to remove excess dye. The formation of red mineralized nodules, a hallmark of osteogenic differentiation, was observed under a TS100 microscope (Nikon).

### Luciferase activity assay

The wild-type (WT) full-length promoter region of the CX43 gene and its site-directed mutagenesis variant (MUT) were cloned into the psiCheck2 reporter vector (Genechem, Shanghai, China) to construct luciferase reporter plasmids for transcriptional activity analysis. HEK 293T cells were co-transfected with the constructed luciferase reporter plasmids and KLF5 overexpression plasmids using Lipofectamine 3000 reagent (Invitrogen, CA, USA) following the manufacturer’s protocol. After 48 h, luciferase activity was assessed using the Dual-Luciferase Reporter Assay System (Promega, WI, USA).

### Chromatin immunoprecipitation (ChIP) assay

SimpleChIP Enzymatic Chromatin IP Kit (Catalog #9003s, Cell Signaling Technology) was used following the provided instructions. Fibroblasts underwent cross-linking with 1% formaldehyde for 10 min, quenching with glycine for 5 min at room temperature, then collected for subsequent steps. Sonication was performed to further fragments the DNA, resulting in extracts ready for immunoprecipitation. The sonicated extracts were incubated with a specific KLF5 antibody (Abcam, USA) or control IgG (Abcam, USA) at 4℃ overnight. Then, it was followed by a reversal of cross-links at 65℃ for 8–10 h to free the associated DNA. The DNA recovered post-immunoprecipitation was then analyzed by PCR to determine the presence and enrichment of target sequences bound by KLF5.

### Statistical analysis

All The analyses were performed using SPSS version 21.0 statistical software (IBM Corp, Armonk, NY), and experimental data are presented as the mean ± SD. The independent samples t-test was used to compare the differences between the two groups. One-way analysis of variance was used to compare more than two groups, followed by Tukey’s post hoc test to account for multiple testing adjustments. A *p* < 0.05 was considered as the threshold for statistical significance.

## Results

### KLF5 expression in OLF and normal LF fibroblasts

**Fig. 1:**
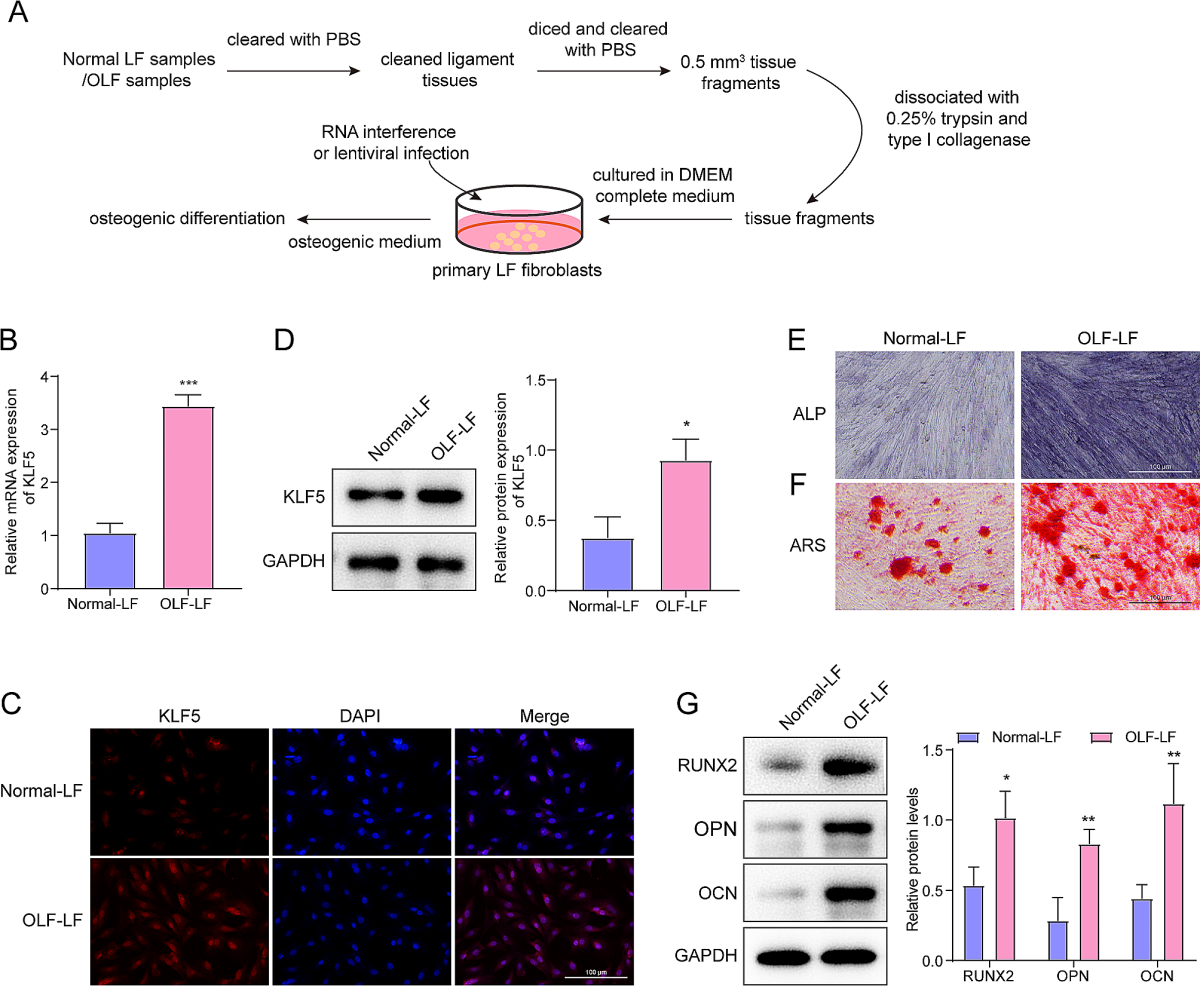
KLF5 expression was upregulated in OLF fibroblasts. **(A)** A flow diagram of primary isolation of clinical samples. **(B)** KLF5 expression in OLF fibroblasts and normal LF fibroblasts was detect by qRT-PCR analysis. **(C)** IF staining was used to detect the KLF5 expression in Normal-LF fibroblasts and OLF fibroblasts (scale bar = 100 μm). **(D)** Western blot was used to detect the protein of KLF5 expression. **(E)** ALP activity was measured using ALP staining (scale bar = 100 μm); **(F)** osteogenic differentiation of OLF fibroblasts was analyzed using ARS staining (scale bar = 100 μm). **(G)** Levels of osteogenic specific markers (RUNX2, OPN, and OCN) were detected using western blot. *p < 0.05, **p < 0.01, ***p < 0.001

We isolated the fibroblasts from the LF and OLF tissues (Fig. 1A). As shown in Fig. 1B, the result of qRT-PCR demonstrated a significant upregulation of KLF5 in OLF fibroblasts. Furthermore, the results of confocal microscopy images showed that KLF5 was mainly localized in the nucleus and more abundantly expressed in OLF fibroblasts compared to normal LF fibroblasts (Fig. 1C). Moreover, the western blot result corroborated the qRT-PCR result confirming enhanced KLF5 expression in OLF fibroblasts (Fig. 1D). In contrast with normal LF fibroblasts, OLF fibroblasts displayed significantly higher osteogenic differentiation, as indicated by ALP and ARS staining (Fig. 1E, F), and the expression changes of the osteogenic transcription factors, RUNX2, and osteoblastic markers, OPN and OCN (Fig. 1G).

### KLF5 silencing effects on OLF fibroblast differentiation

**Fig. 2:**
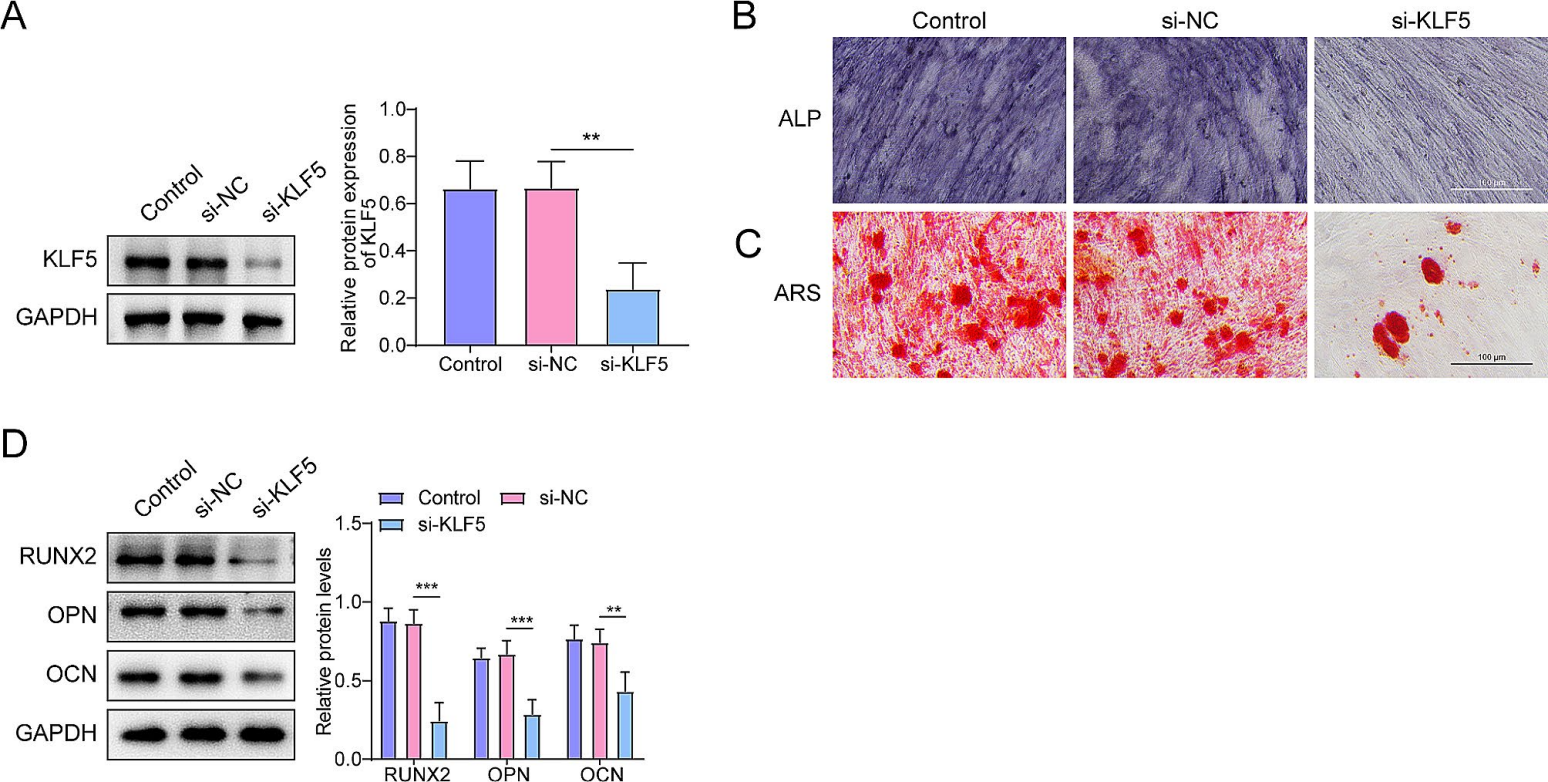
KLF5 knockdown significantly decreased the osteogenic ability of OLF fibroblasts **(A)** The silencing effect of siRNA on KLF5 was measured using western blot in OLF fibroblasts. **(B)** ALP activity was measured using ALP staining (scale bar = 100 μm). **(C)** Osteogenic differentiation of OLF fibroblasts was measured using ARS staining (scale bar = 100 μm). **(D)** Levels of osteogenic specific markers (RUNX2, OPN, and OCN) were detected using western blot. **p < 0.01, ***p < 0.001

The decrease in KLF5 expression of the si-KLF5 group of OLF fibroblasts was confirmed by western blot analysis (Fig. 2A). We found that KLF5 suppression reduced osteogenic differentiation, evident from the lower ALP activity in the si-KLF5 group compared to the si-NC group (Fig. 2B). ARS analysis showed that si-KLF5 significantly decreased the area of nodules of OLF fibroblasts (Fig. 2C). The knockdown of KLF5 also led to a lower expression of osteogenic markers, including RUNX2, OPN, and OCN, in the OLF fibroblasts (Fig. 2D), highlighting the importance of KLF5 in the osteogenic programming of OLF fibroblasts.

### KLF5 overexpression promotes osteogenic differentiation of LF fibroblasts

**Fig. 3:**
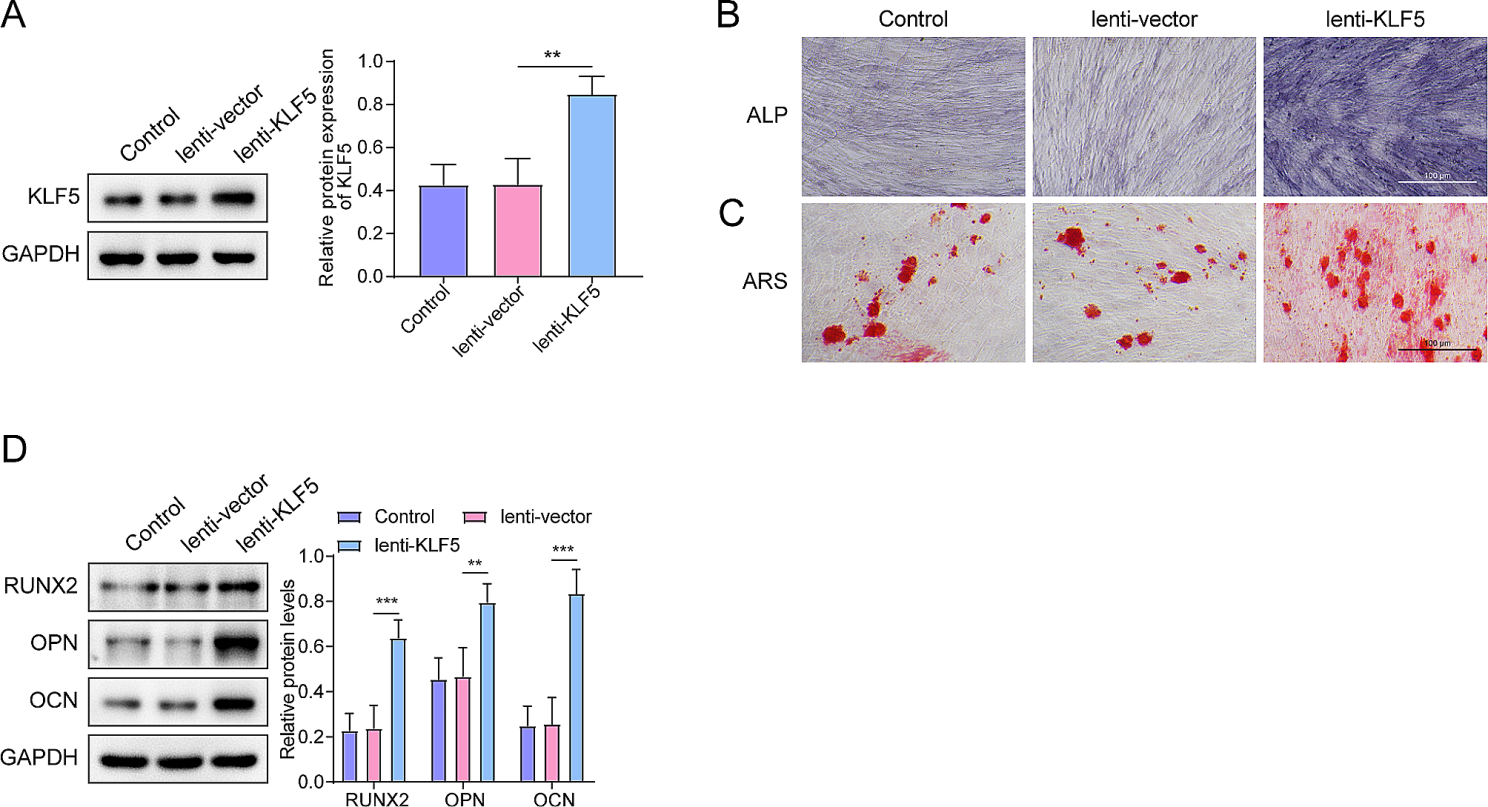
Overexpression of KLF5 significantly increased the osteogenic ability of normal LF fibroblasts. **(A)** The effect of lenti-KLF5 in normal LF fibroblasts was measured using western blot. **(B)** ALP activity was measured using ALP staining (scale bar = 100 μm). **(C)** Osteogenic differentiation of normal-LF fibroblasts was measured using ARS staining (scale bar = 100 μm). **(D)** Levels of osteogenic specific markers (RUNX2, OPN, and OCN) were detected using western blot. **p < 0.01, ***p < 0.001

Normal LF fibroblasts were infected with the lentivirus, and successful overexpression was verified via western blot analysis, demonstrating effective transfection (Fig. 3A). Increased KLF5 expression resulted in enhanced ALP activity and greater nodule formation area, indicative of osteogenic differentiation (Fig. 3B, C). We found that overexpression of KLF5 upregulated the levels of key osteoblastic markers, such as RUNX2, OPN, and OCN in OLF fibroblasts (Fig. 3D). Taken together, KLF5 promotes osteogenic differentiation of LF fibroblasts.

### KLF5 directly activates CX43 transcription

**Fig. 4:**
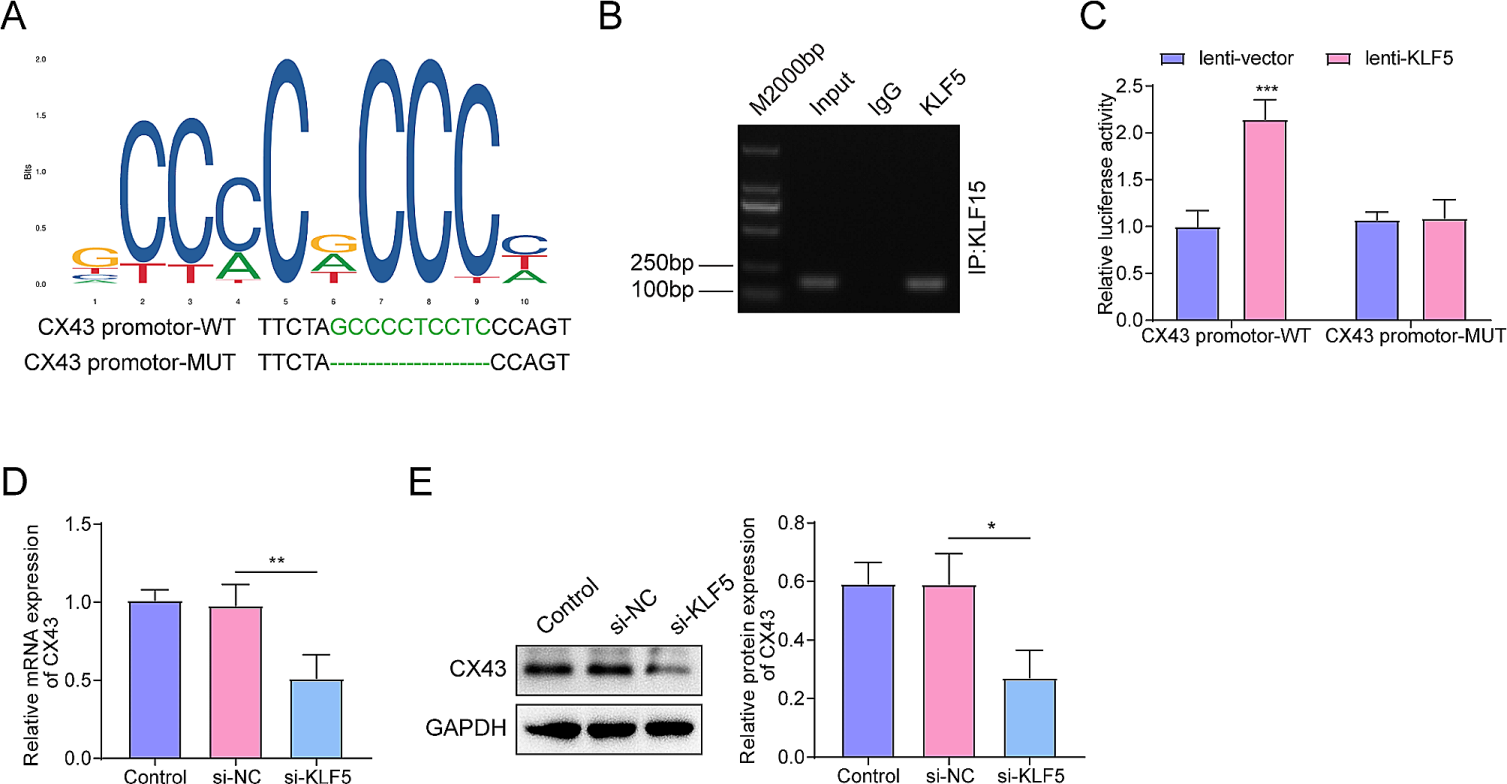
CX43 is a direct transcriptional target of KLF5. **(A)** The transcriptional factor KLF5 binding motif from the JASPAR database. **(B)** The ChIP-PCR assay verified the direct binding of KLF5 and CX43 promoter in OLF fibroblasts. **(C)** Dual-luciferase reporter assays on the wild-type and mutated CX43 promoters. **(D)** Effect of si-KLF5 on mRNA level of CX43 was measured using RT-qPCR. **(E)** Effect of si-KLF5 on the protein level of CX43 was measured using western blot. *p < 0.05, **p < 0.01, ***p < 0.001

This search yielded a highly conserved KLF5 binding sequence ranging from − 63 bp to -54 bp upstream of the transcription start site of CX43(Fig. 4A). ChIP-PCR analysis with an anti-KLF5 antibody (and normal rabbit IgG as a control) corroborated the interaction between KLF5 and the CX43 promotor (Fig. 4B). Luciferase reporter assays showed that mutation of this KLF5 binding site significantly reduced the KLF5-induced transactivation of the CX43 promoter (Fig. 4C). The downregulation of KLF5 in OLF fibroblasts by the si-KLF5 decreased the expression of CX43 (Fig. 4D and E). These results indicated that KLF5 could directly activate CX43 transcription.

### KLF5 promotes osteogenic differentiation of OLF fibroblasts by regulating CX43

**Fig. 5:**
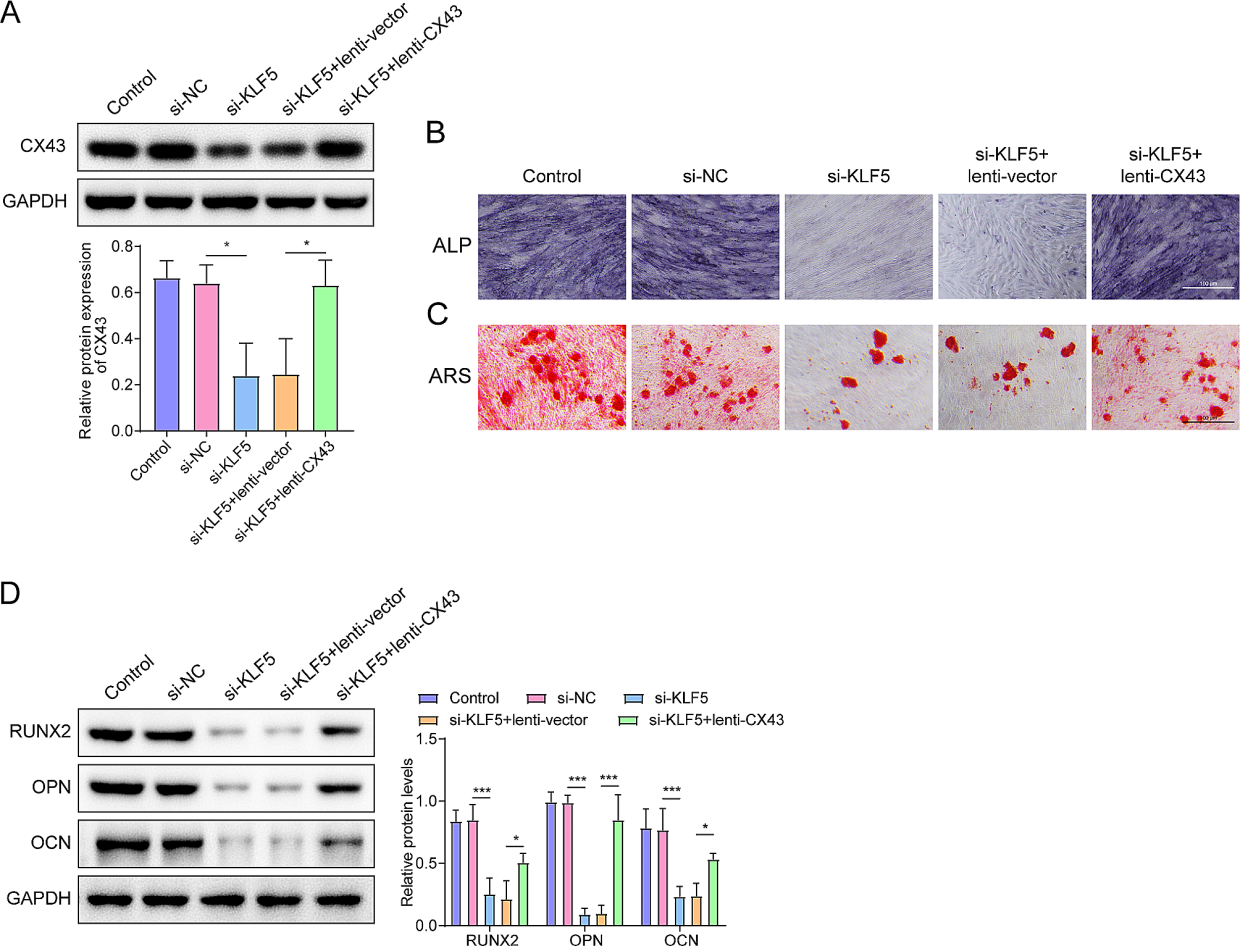
KLF5 targeted CX43 to promote osteogenic differentiation of OLF fibroblasts. **(A)** Protein expression levels of CX43 after OLF fibroblasts after the intervention of KLF5 and overexpression of CX43. **(B)** ALP activity was measured using ALP staining (scale bar = 100 μm). **(C)** Osteogenic differentiation of OLF fibroblasts was measured using ARS staining (scale bar = 100 μm). **(D)** Levels of osteogenic specific markers (RUNX2, OPN, and OCN) were detected using western blot. *p < 0.05, ***p < 0.001

The protein expression of CX43 is shown in Fig. 5A. The silencing of KLF5 appeared to impair the differentiation potential of OLF fibroblasts, as ALP activity and the area of nodules was reduced. However, overexpression CX43 rescued the osteogenic ability suppressed by si-KLF5 (Fig. 5B, C). Consistent with these results, si-KLF5 also led to attenuated induction of several osteogenic markers, such as RUNX2, OPN, and OCN, in OLF fibroblasts. At the same time, these effects were counteracted by CX43 overexpression (Fig. 5D). In summary, KLF5 silencing hampers the osteogenic process in LF. In contrast, CX43 overexpression can counterbalance this inhibition, underscoring that KLF5 promotes osteogenic differentiation of OLF fibroblasts by regulating CX43.

## Discussion

Ossification of the LF is a pathological condition characterized by the transformation of the LF from a soft, flexible tissue to one that is hardened or ossified. This condition often results in severe neurological deficits and poor outcomes for patients, attributed to its intricate pathophysiology and the various contributing factors [23, 24]. Histologically, OLF is initiated by chondroid metaplasia followed by an infiltration of fibroblast-like mesenchymal cells into the ligament matrix. The abnormal or increased osteogenic differentiation contributes to the overgrowth of bone in the LF [25]. This dysregulation can lead to the production of excessive bone matrix and mineralization, eventually resulting in ossification [8]. Previous studies have reported that crucial molecular players involved in osteogenic differentiation [26], such as RUNX2, osterix (OSX), bone morphogenetic proteins (BMPs), and Wnt/β-catenin signaling pathways, could potentially be correlated with OLF [25, 27]. However, the mechanism driving OLF remains to be fully delineated. This study is the first to document that KLF5 is a significant facilitator of osteogenesis in OLF fibroblasts by upregulating CX43 expression at the transcriptional level. Our findings add a novel dimension to the molecular understanding of OLF and lay the groundwork for future research into its therapeutic targeting.

KLF5 is a transcription factor that plays an important role in osteogenic differentiation [28]. The role of KLF5 in osteogenic differentiation has been reported in the osteogenesis of human periodontal ligament fibroblasts [13]. In this study, we initially observed a noteworthy elevated level of KLF5 expression amidst OLF fibroblasts compared to normal LF fibroblasts. Furthermore, unique osteogenic differentiation markers like RUNX2 [29], OPN [30, 31], and OCN [32] were also upregulated in OLF fibroblasts. Our findings suggest that osteogenic differentiation plays a pivotal role in the pathology of OLF. Delving into the function of KLF5 within this process, our results indicate that silencing KLF5 with siRNA diminishes nodule formation and impairs the osteogenic potential of OLF fibroblasts. Furthermore, the downregulation of KLF5 coincides with decreased expression levels of key osteogenic markers, including Runx2, OPN, and OCN, during the differentiation process. Contrastingly, an upregulation or overexpression of KLF5 in normal LF fibroblasts led to a substantial surge in mineralization and, subsequently, overall facilitation of osteogenic differentiation. This tit-for-tat relationship between KLF5 expression and osteogenic differentiation potential indicated that KLF5 acts as a critical OLF biomarker.

Connexin 43 (CX43) actively contributes to the generation and homeostasis [33, 34]. This nexus drew attention as CX43 is well-known for bone remodeling and healing due to its regulation of osteoclast and osteoblast functions [21, 35, 36]. A previous study indicated that CX43 promotes OLF through the p38 MAPK-RUNX2 signaling pathway [21]. Intriguingly, we observed a link between KLF5 and CX43. Our data showed that KLF5 directly activates CX43 transcription. Encouragingly, over-expressed CX43 could rescue the suppressed differentiation by KLF5, elucidating the crucial role of the KLF5-CX43 pathway in osteogenic differentiation.

## Conclusion

In conclusion, our results reveal that KLF5 promotes OLF by transcriptionally activating CX43. While these findings advance our understanding of OLF pathobiology, they also pave the way for future investigations into the specific role of this KLF5-CX43 pathway, presenting new therapeutic approaches for OLF and possible bone-related disorders. There are also some limitations of this study. The in-depth mechanism of the KLF5-CX43 axis yet to be fully clarified. Additionally, whether the KLF5/CX43 axis can be employed as a breakthrough point for OLF treatment needs further study.

## Data Availability

No datasets were generated or analysed during the current study.
